# A new regulatory mechanism controlling carotenogenesis in the fungus *Mucor circinelloides* as a target to generate β-carotene over-producing strains by genetic engineering

**DOI:** 10.1186/s12934-016-0493-8

**Published:** 2016-06-07

**Authors:** Yingtong Zhang, Eusebio Navarro, José T. Cánovas-Márquez, Lorena Almagro, Haiqin Chen, Yong Q. Chen, Hao Zhang, Santiago Torres-Martínez, Wei Chen, Victoriano Garre

**Affiliations:** State Key Laboratory of Food Science and Technology, School of Food Science and Technology, Jiangnan University, Wuxi, 214122 People’s Republic of China; Departamento de Genética y Microbiología (Associate Unit to IQFR-CSIC), Facultad de Biología, Universidad de Murcia, 30100 Murcia, Spain; Beijing Innovation Centre of Food Nutrition and Human Health, Beijing Technology and Business University (BTBU), Beijing, 100048 People’s Republic of China; Department of Plant Biology, Faculty of Biology, University of Murcia, Campus de Espinardo, 30100 Murcia, Spain

**Keywords:** β-carotene, Carotenogenesis regulation, Whole-genome sequencing, Knockout mutants, *crgA*

## Abstract

**Background:**

Carotenoids are natural pigments with antioxidant properties that have important functions in human physiology and must be supplied through the diet. They also have important industrial applications as food colourants, animal feed additives and nutraceuticals. Some of them, such as β-carotene, are produced on an industrial scale with the use of microorganisms, including fungi. The mucoral *Blakeslea trispora* is used by the industry to produce β-carotene, although optimisation of production by molecular genetic engineering is unfeasible. However, the phylogenetically closely related *Mucor circinelloides,* which is also able to accumulate β-carotene, possesses a vast collection of genetic tools with which to manipulate its genome.

**Results:**

This work combines classical forward and modern reverse genetic techniques to deepen the regulation of carotenoid synthesis and generate candidate strains for biotechnological production of β-carotene. Mutagenesis followed by screening for mutants with altered colour in the dark and/or in light led to the isolation of 26 mutants that, together with eight previously isolated mutants, have been analysed in this work. Although most of the mutants harboured mutations in known structural and regulatory carotenogenic genes, eight of them lacked mutations in those genes. Whole-genome sequencing of six of these strains revealed the presence of many mutations throughout their genomes, which makes identification of the mutation that produced the phenotype difficult. However, deletion of the *crgA* gene, a well-known repressor of carotenoid biosynthesis in *M. circinelloides*, in two mutants (MU206 and MU218) with high levels of β-carotene resulted in a further increase in β-carotene content to differing extents with respect to the *crgA* single-null strain; in particular, one strain derived from MU218 was able to accumulate up to 4 mg/g of β-carotene. The additive effect of *crgA* deletion and the mutations present in MU218 suggests the existence of a previously unknown regulatory mechanism that represses carotenoid biosynthesis independently and in parallel to *crgA*.

**Conclusions:**

The use of a mucoral model such as *M. circinelloides* can allow the identification of the regulatory mechanisms that control carotenoid biosynthesis, which can then be manipulated to generate tailored strains of biotechnological interest. Mutants in the repressor *crgA* and in the newly identified regulatory mechanism generated in this work accumulate high levels of β-carotene and are candidates for further improvements in biotechnological β-carotene production.

**Electronic supplementary material:**

The online version of this article (doi:10.1186/s12934-016-0493-8) contains supplementary material, which is available to authorized users.

## Background

β-carotene is a fat-soluble pigment that has several biological functions and great commercial value due to its diverse uses in food, pharmaceutical products, cosmetics and textiles [1–3]. Thus, the global carotenoid market value was $1.5 billion in 2014 and is expected to reach nearly $1.8 billion in 2019, with a compound annual growth rate of 3.9 % [4]. Microbial β-carotene has attracted much attention because of its rapid generation and lack of influence from geographic location and climate. The microbial β-carotene currently produced by the industry comes from the alga *Dunaliella salina* [5] or the fungus *Blakeslea trispora* [6]. *B. trispora* accumulates large amounts of carotenoids, as do other fungi of the subphylum mucormycotina, but the absence of molecular tools with which to manipulate its genome makes it difficult to improve carotenoid production. In contrast, the genome of *Mucor circinelloides*, which belongs to the same subphylum, has been sequenced (http://www.genome.jgi.doe.gov/Mucci2/Mucci2.home.html), and procedures for efficient genetic transformation, gene replacement and gene silencing by RNAi [7] allow improvement of carotene production by genetic manipulation.

Fungal carotenoids derive from 3-hydroxy-3-methyl-glutaryl-CoA (HMG-CoA) in the mevalonate pathway [8] and is regulated by complex genetic mechanisms. Light induction of carotenoid biosynthesis is considered to be conserved in filamentous fungi such as *Neurospora crassa*, *Phycomyces blakesleeanus* and *M. circinelloides* [9–11]. Transcription of structural genes for carotenoid biosynthesis increased enormously in response to light, in accordance with the improved carotene content. In the case of *N. crassa*, a model was established in which induction of carotenogenesis by light is mediated by the white-collar protein complex that is formed with the products of *wc*-*1* and *wc*-*2* genes [12, 13]. Genes homologous to *wc*-*1* and *wc*-*2* have been identified in several fungi, and one of the three homologs to *wc*-*1* of *M. circinelloides*, *mcwc*-*1c*, was also shown to be involved in the induction of carotenogenesis by light [9]. Deletion of *mcwc*-*1c* led to only a threefold increase in β-carotene levels, compared to the 21-fold increase in the wild-type strain after light exposure, which indicates that light-induced carotenogenesis in *M. circinelloides* requires *mcwc*-*1c* [9].

Another regulatory gene identified in *M. circinelloides* is *crgA*, which codes for a RING finger protein that was regarded as a negative regulator of carotenoid biosynthesis, because its disruption resulted in β-carotene overproduction in the dark as a result of a sharp increase of the *carB* and *carRP* mRNA levels [14]. The presence of RING-finger zinc-binding domains, which define a family of ubiquitin ligases that mediate ubiquitylation of target proteins, suggests that CrgA acts as an E3 ubiquitin ligase. In fact, CrgA blocks the function of Mcwc-1b, a protein encoded by a second *wc*-*1* homolog, by mono-ubiquitylation and di-ubiquitylation without degradation [15], which has also been observed in the regulatory process of the budding yeast transcription factor Met4 [16].

The *crgA*-mediated repression of carotenogenesis and light-induced carotenogenesis are independent regulatory pathways because the *crgAmcwc*-*1c* double mutant presented similar levels of β-carotene as the *crgA* single mutant in the dark but was not able to enhance the production to the same level as the *crgA* single mutant after illumination [15]. The occurrence of multiple copies of *wc*-*1* genes in mucoromycotina fungi such as *M. circinelloides* [9], *P. blakesleeanus* [17], *Rhizopus oryzae* [17] and *Pilobolus crystallinus* [18], and the existence of four transduction pathways in charge of different light responses in *P. blakesleeanus,* suggests that these basal fungi may have developed a more complex light regulatory system than dikaryotic fungi (ascomycetes and basiodiomycetes), which contains a single copy of the *wc*-*1* gene [19, 20]. Moreover, the abundance of genes that regulate carotenogenesis, such as *carC*, *carD*, *carS*, *carF* and *carI* in *P. blakesleeanus* [21–24], suggests that carotene biosynthesis in *M. circinelloides* may be also regulated by a variety of elements.

In this report, we analysed 34 mutants in which carotenoid biosynthesis was affected to examine the regulatory mechanisms that control carotenoid biosynthesis in *M. circinelloides* and to generate an overproducing strain of interest for β-carotene production. Eight of these mutants lacked mutations on known structural or regulatory genes, suggesting that they contained mutations in unknown genes involved in carotenoid biosynthesis. Two of these mutants, MU206 and MU218, clearly carried mutations in regulatory genes because they accumulated greater amounts of β-carotene than the wild-type strain. Deletion of *crgA* in both strains produced an increase in β-carotene levels that was much greater in the *crgA*-null derivative of MU218, which suggests that the gene affected in MU218 is involved in a regulatory pathway other than that of *crgA*, whereas the gene mutated in MU206 may participate in the same regulatory pathway as *crgA.* Moreover, some of the overproducing strains may be of interest for industrial production of β-carotene.

## Results

### Isolation of mutants affected in the regulation of carotenoid biosynthesis

The mycelium of *M. circinelloides* has a white-yellowish appearance in the dark because it accumulates a small amount of β-carotene. In the light, the biosynthesis of this pigment is stimulated and the mycelium becomes deep yellow. To identify new genes implicated in the regulation of carotenogenesis, mutagenized spores of the wild-type strain R7B were allowed to follow a complete vegetative cycle to permit expression of recessive mutations, since initial mutants are heterokaryons due to the multinucleate nature of R7B. The recycled spores were used to screen for colour mutants both in the dark and after exposure to light.

After screening about 4 × 10^5^ colonies, 24 colour mutants from N-methyl-N’-nitro-N-nitrosoguanidine (NTG) mutagenesis were isolated and purified by successive vegetative cycles until all of the colonies from each particular mutant showed the very same colour phenotype, indicating that they were true homokaryotic mutants. The strains were named from MU260 to MU269, MU271 to MU278 and MU280 to MU285. After ultraviolet (UV) light treatment, only two colour mutants, MU279 and MU286, were isolated after screening about 5.6 × 10^5^ spores. In this study, we included mutants (MU206, MU212 to MU218) from our collection (University of Murcia, Spain) that were isolated during a previous screening of colonies derived from a different NTG treatment [25]. Most of the mutants showed a white colour in the dark as the wild-type strain but appeared white or pale yellow in the light, whereas the wild-type strain showed a yellow colour (Table 1). In contrast, mutant MU206 and MU218 appeared pale yellow in the dark and yellow and deep yellow, respectively, in the light.

**Table 1:** Mutants in carotenoid biosynthesis analysed in this work

Strain	Color in the dark	Color in the light	Mutation in carotenogenic genes
R7B (wild-type)	White	Yellow	None
MU206	Pale yellow	Yellow	None
MU212, 213, 214	White	White	5′ splice site intron 1 *mcwc1c*
MU215	White	White	5′ splice site intron 4 *mcwc1c*
MU216	White	Pale yellow	None
MU217	White	Pale yellow	None
MU218	Pale yellow	Deep yellow	None
MU260	White	White	D53G CarB
MU261	White	White	C436R CarRP
MU262	White	White	T308 M CarB
MU263	White	White	20 bp deletion *carB*
MU264	White	White	None
MU266	White	Pale yellow	S317F CarB
MU265, MU267, MU280	White	Pale yellow	K143E Mcwc1c
MU268, MU269, MU271, MU272	White	Pale yellow	S77P Mcwc1c
MU273, MU276,	White	White	G433D CarRP
MU274, MU275, MU277, MU284	White	White	C235R CarRP
MU278	White	White	Deletion in region *carB*-*carRP*
MU279^a^	White	White	Deletion in region *carB*-*carRP*
MU281	White	White	S302F CarRP
MU282	White	Pale yellow	None
MU283	White	Pale yellow	None
MU285	White	Pale yellow	None
MU286^a^	White	Pale yellow	None

^a^MU279 and MU286 derived from UV-treated spores whereas the remaining mutants came from treatment with NTG

### Analysis of carotenogenic genes in mutants

The phenotypes shown by the mutants could be due to mutations in the structural carotenogenic genes (*carB* and *carRP*) and in known (*mcwc*-*1c*) [9] or unknown regulatory genes, including the genes that code for putative homologs to *N. crassa wc*-*2* [26] and *P. blakesleeanus madB* [17]. Mutations in the repressor *crgA* were not considered because none of the mutants showed a bright yellow colour, which is a hallmark of mutations in that gene [14]. Thus, the *carB*-*carRP* genomic region, including coding and promoter sequences, and coding and promoter sequences (~200 bp) of *mcwc*-*1c* and the four putative *mcwc*-*2* genes (ID78608, ID85699, ID153812 and ID157130) of the 34 colour mutants were amplified, and the polymerase chain reaction (PCR) fragments were directly sequenced without previous cloning to avoid detection of mutations produced by the PCR. The obtained sequences were compared to the corresponding wild-type alleles of the reference genome (http://www.genome.jgi.doe.gov/Mucci2/Mucci2.info.html) to identify mutations. The results of this analysis show that 25 strains presented different mutations in *mcwc1c*, *carB* and *carRP*, including deletions of *carB*-*carRP*, characterised by Southern blot hybridisation (data no shown), in two strains (Table 1). Several mutants carried the same mutation suggesting that they derived from an original mutant that was amplified in the spore-recycling step. Surprisingly, no mutations were found in the four *mcwc*-*2* genes. In contrast, nine mutants showed no mutations in the analysed genes and should thus carry mutations in uncharacterised genes that are probably involved in the regulation of carotenoid biosynthesis.

### Carotene content in carotenogenic mutants with uncharacterised mutations

To confirm the phenotype of the nine mutants with no mutations in known carotenogenic and putative regulatory genes, their carotene content was determined both in the dark and light and compared with that of the wild-type parental strain R7B. Five of the six strains with a pale yellow colour in light accumulated lower amounts of carotenes than the wild-type strain (Table 2), whereas MU282 showed β-carotene levels similar to those of the wild-type strain even though its mycelium was pale yellow in light. The discrepancy between colour and β-carotene content in this mutant could be caused by a growth defect because its mycelium was less dense than that of the wild-type strain. Scanning spectrophotometry of the carotenoids accumulated in light by the pale yellow mutants showed the characteristic shape and peaks for β-carotene, indicating that this is the main carotene accumulated by all of them, as it happens in the wild-type strain (Additional file 1: Figure S1). The accumulation of mainly β-carotene in the wild-type strain was confirmed by high-performance liquid chromatography (Additional file 2: Figure S2).

**Table 2:** Amount of β-carotene accumulated by mutants affected in carotenoid biosynthesis

Strain	β-carotene (μg/g dry mass)	
	Dark	Light
Wild type (R7B)	8.6 ± 2.6^ab^	170.8 ± 11.9^d^
MU206	81.6 ± 9.4^d^	254.7 ± 24.1^e^
MU216	4.8 ± 0.4^ab^	119.7 ± 16.9^c^
MU217	6.0 ± 0.6^ab^	75.7 ± 8.1^b^
MU218	63.1 ± 6.3^c^	736.1 ± 20.3^f^
MU264	0.0 ± 0.0^a^	0.0 ± 0.0^a^
MU282	8.2 ± 0.2^ab^	165.8 ± 8.5^d^
MU283	5.5 ± 0.8^ab^	71.3 ± 11.5^b^
MU285	12.0 ± 2.0^b^	54.8 ± 5.5^b^
MU286	8.9 ± 1.6^ab^	47.2 ± 9.0^b^

^a–f^Values are mean ± standard deviation of at least three independent experiments and values within a column with different superscript letters were significantly different (p < 0.05)

In contrast, the white mutant MU264 showed undetectable levels of β-carotene in the dark and light (Table 2; Additional file 1: Figure S1). Special attention was dedicated to this mutant and carotenoids accumulated by this mutant were analysed by HPLC (Additional file 2: Figure S2). No carotenoids were detected in light, including phytoene, indicating that MU264 carries a mutation that blocks completely the carotenoid pathway. In addition to the mutants with low β-carotene levels, two strains, MU206 and MU218, accumulated high levels of β-carotene both in the dark and in light, suggesting that they are affected in genes involved in the regulation of carotenoid biosynthesis. The levels of β-carotene in those strains in the dark were roughly ten times higher than the levels in the wild-type strain R7B, whereas increases in light of 1.5 and 4.5 times were seen in MU206 and MU218, respectively (Table 2).

### Characterisation of carotenogenic mutant genomes

The devised strategy for identification of mutations that provoked alterations in β-carotene levels was sequencing of the entire genome followed by comparison to the *M. circinelloides* reference genome. Although eight strains showed altered β-carotene levels, six strains (MU206, MU216, MU217, MU218, MU264 and MU286) with different pattern of β-carotene accumulation were selected for whole-genome sequencing and two (MU283 and MU285) were preserved for future analyses. In addition to the six strains with altered carotenogenesis, the genome of parental strain R7B was sequenced for use as a reference because this strain was obtained by UV light treatment and could harbour additional mutations to that in the 3-isopropylmalate dehydratase gene (*leuA*) gene that makes it auxotrophic for leucine [27].

The genome sequences of the seven genomes were obtained using SOLiD technology, and the sequences were mapped against the *M. circinelloides* reference genome (http://www.genome.jgi.doe.gov/Mucci2/Mucci2.info.html) to detect base substitutions and small deletions and insertion (indels). More than 99.7 % of the reference genome was successfully mapped for each sample, and the mean coverage depth over that mapped region ranged from 39.8 to 53.6× (Additional file 3: Table S1). As a proof of the quality of the genome sequences, the known single-base substitution in the *leuA* gene that causes a change from glutamic acid to lysine at position 220 [28] was detected in all genomes (Additional files 4, 5: Tables S2, S3). As expected, the R7B genome contained additional mutations, which included three base substitutions that resulted in amino acid changes in a putative E3 ubiquitin-protein ligase (P111S), in a DUF1688 domain–containing protein (D331Y) and in a putative helicase-2 (S308G) (Additional files 4, 5: Tables S2, S3). In addition, 11 base substitutions were found in intergenic regions, one in the intron 1 of basic helix-loop-helix transcription factor-like protein (ID113859) and two others that did not produce amino acid changes (ID76084 and ID164535). Each of these base substitutions was present in the six mutants, except the mutation in the DUF1688 domain–containing protein and two base substitutions in intergenic regions that only appear in MU264 and MU286. The strains without those substitutions were obtained from spores treated with NTG in 1993 and kept frozen until 2009, whereas the strains with the substitutions were isolated in 2009. The genomic DNA from R7B was isolated from the strain used in 2009 and, therefore, it is possible that those changes occurred spontaneously in the R7B strain in our laboratory between 1993 and 2009. In any case, the presence of the mutation in the gene coding for the DUF1688 domain–containing protein in R7B discarded the notion that this mutation is responsible for the phenotype of the mutants.

All mutant strains showed a large number of base substitutions and some indels in comparison to R7B (Table 3; Additional files 4, 5, 6: Tables S2, S3, S4), and most of them corresponded to intergenic regions. Only missense mutations or indels in coding regions were considered to be causative for the phenotypes displayed by the mutants and were analysed in detail. The mutant MU206 presented just one missense mutation (A125 V) in a predicted gene that codes for a protein that only appears in *M. circinelloides* (Table 3). MU264 carries two missense mutations: one (D380 N) in the gene *aspB* that codes for a putative septin and the other one (C171Y) in a gene that codes for a putative F-box protein (Table 3). The mutation in the F-box protein affected one of the crucial residues of the F-box domain. In addition to the missense mutations, MU264 has an 11-bp deletion in a gene that codes for a class 3 adenylyl cyclase that provokes truncation of the protein (Table 3). MU286 has one missense mutation (P152L) in a gene that codes for a class 3 lipase and two small deletions that originate truncated proteins, one in a gene that codes for a protein similar to a vacuolar dynamin-like GTPase VpsA and another in a gene that codes for a protein similar to the autophagy-related protein 9 (Table 3). Mutants MU216, MU217 and MU218 have many mutations in several genes (Table 3), and it is difficult to determine which of them is responsible for the mutant phenotype.

**Table 3:** Mutations present in the carotenogenic mutants

Strain	Mutation	ID	Gene
R7B	Scaffold 2: 2919466 Scaffold 4: 538767 Scaffold 11: 599712 Scaffold 6: 2888792	33992 162470 86468 165433	3-isopropylmalate dehydratase E3 ubiquitin ligase Helicase 2 DUF1688
MU206	Scaffold 2: 3372803	107362	Predicted protein
MU216	Many	Many	Many
MU217	Scaffold 1: 2734563 Scaffold 1: 3851481 Scaffold 1: 5070681 Scaffold 1: 5222094 Scaffold 1: 5816574 Scaffold 2: 2533223 Scaffold 3: 2140181 Scaffold 3: 2748240 Scaffold 4: 2327078 Scaffold 4: 2465882 Scaffold 5: 541005 Scaffold 5: 2147324 Scaffold 6: 389132 Scaffold 6: 2797394 Scaffold 7: 408056 Scaffold 8: 1129513 Scaffold 11: 585629	105120 158722 31540 105956 176325 160164 161513 155773 156146 81849 162471 129474 164712 112987 180434 157185 150394	Cdc4 and related F-box and WD-40 proteins Similar to dentin sialophosphoprotein Hypothetical protein (WD40 domain) Hypothetical protein (PX domain) Type IA hybrid histidine kinase Predicted protein DEAD/DEAH box DNA helicase (Mer3) Phytochelatin synthase Putative DNA-binding protein Predicted protein Serine/threonine protein kinase Transcription factor Chromodomain helicase DNA binding protein DNA excision repair protein (Rad5) Glutamine-fructose-6-phosphate transaminase Autophagic serine protease Alp2 Exocyst comlex component Sec10
MU218	Scaffold 1: 1190571 Scaffold 1: 3673453 Scaffold 1: 4109183 Scaffold 2: 460034 Scaffold 2: 46601146 Scaffold 3: 1851291 Scaffold 3: 4242409, 4243539 Scaffold 4: 580831 Scaffold 4: 647221 Scaffold 4: 3597815 Scaffold 5: 2893384 Scaffold 6: 218062 Scaffold 6: 835657 Scaffold 6: 2366334 Scaffold 7: 1528520 Scaffold 7: 2134543 Scaffold 8: 1828638 Scaffold 9: 869061 Scaffold 9: 965023 Scaffold 10: 586865	136827 158673 76500 155155 107817 108531 162134 162480 80915 143638 82957 75161 112339 41089 119794 75421 148442 167359 85497 115432	Mitocondrial carrier protein Nuclear distribution protein NUDC Related to plant expansions Amido phosphoribosyltransferase E3 Ring finger ubiquitin ligase Phospholipid/glycerol acyltransferase E3 Ubiquitin ligase (HECT) Basic-leucine zipper (bZIP) transcription factor Predicted protein DnaJ domain protein WD repeat-containing protein Golgi SNAP receptor complex member Thioesterase/thiol ester dehydrase-isomerase Sec5 subunit of exocyst complex Kinesin like protein Iron/zinc ion transporter Translation elongation factor EF1B Predicted protein Zinc finger, PHD-type Hypothetical protein
MU264	Scaffold 1: 1206002 Scaffold 4: deletion 1887965–1893565 Scaffold 6: 2888792 Scaffold 7: 2443177	104636 81312 165433 166227	Septin family protein Adenylyl cyclase class 3–4/guanylyl cyclase DUF1688 F-box protein containing LRR
MU286	Scaffold 5: 2245854 Scaffold 1: deletion 635987–635988 Scaffold 3: deletion 4425722–4425732 Scaffold 5: 2245854 Scaffold 6: 2888792	111734 157796 116964 111734 165433	Lipase class 3 Vacuolar sorting protein VPS1 Autophagy-related protein 9 Lipase class 3 DUF1688

To validate the whole-genome sequencing, affected genes from mutants with the fewest affected genes (MU206, MU264 and MU286) were amplified by PCR and sequenced. In all cases, the sequences of the amplified fragments contained the mutations identified in whole-genome sequencing (data not shown), validating the whole-genome sequencing. Moreover, those mutants were transformed with self-replicative plasmids that contained the wild-type alleles corresponding to the mutated genes, and functional complementation was not observed in any case (data not shown), which suggests either the genes in the plasmids were not efficiently expressed or that the phenotype could be provoked for mutations in putative intergenic regions. The lack of complementation is particularly relevant in the case of mutant MU206 because it showed just one base substitution in one gene and was used as a parental strain in the experiments described below.

### Generation of *crgA* null mutants in MU206 and MU218 backgrounds

**Fig. 1:**
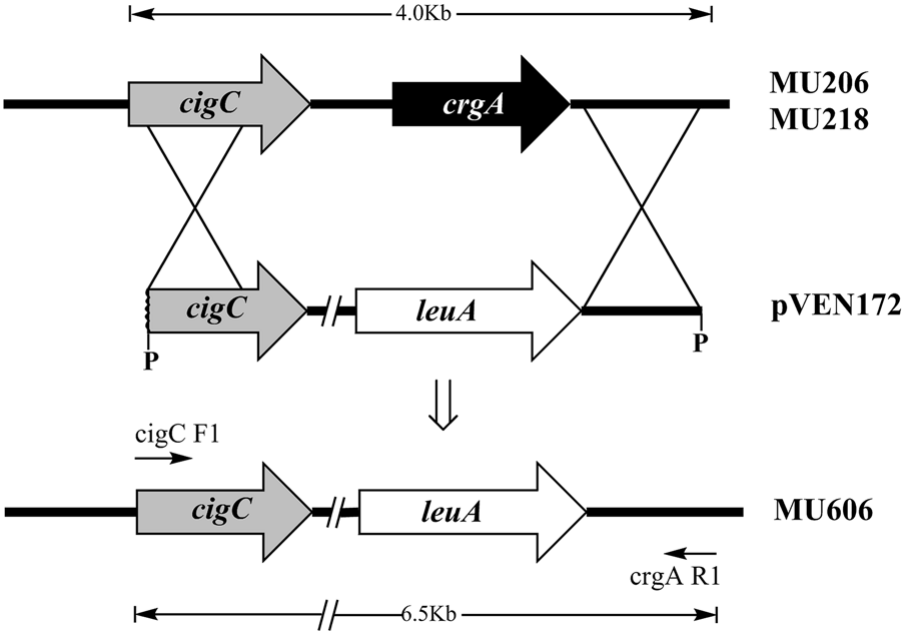
The genomic structure of *crgA* wild-type locus and after disruption by homologous recombination. The replacement fragment used to disrupt *crgA* gene corresponded to a 6.5-kb *Pst*I fragment derived from plasmid pVEN172. The positions of primers CigC F1/crgA R1 used to amplify the disrupted and wild-type *crgA* locus, and the expected *sizes* of the corresponding PCR products, are indicated

The enhancement of carotene biosynthesis in MU206 and MU218 (Table 2) suggests that they were affected in genes involved in some regulatory pathway that controls carotenoid biosynthesis. This hypothetical pathway might limit the accumulation of carotenoid in the dark, as with the regulatory pathway in which the *crgA* gene is the principal element [14, 15]. The lack of the *crgA* gene led to enrichment of carotene in both dark and light conditions [14], as with MU206 and MU218, although they lacked mutations in the *crgA* gene (Table 3). Disruption of the *crgA* gene in these two strains could affect carotenogenesis regulation, in addition to generation of β-carotene–overaccumulating strains of potential interest for industrial production of β-carotene. For this purpose, plasmid pVEN172 [14], which harbours the *leuA* wild-type allele gene of *M. circinelloides* flanked by the sequences neighbouring the *crgA* gene, was adopted in this study. The 6.5-kb fragment released by digestion of pVEN172 with restriction enzyme *Pst*I was used to transform MU206 and MU218, both of which are *leuA* auxotrophs. Integration of this fragment into the genome of the recipient strains by homologous recombination was expected to cause replacement of the endogenous *crgA* gene with the wild-type *leuA* allele gene (Fig. 1).

**Fig. 2:**
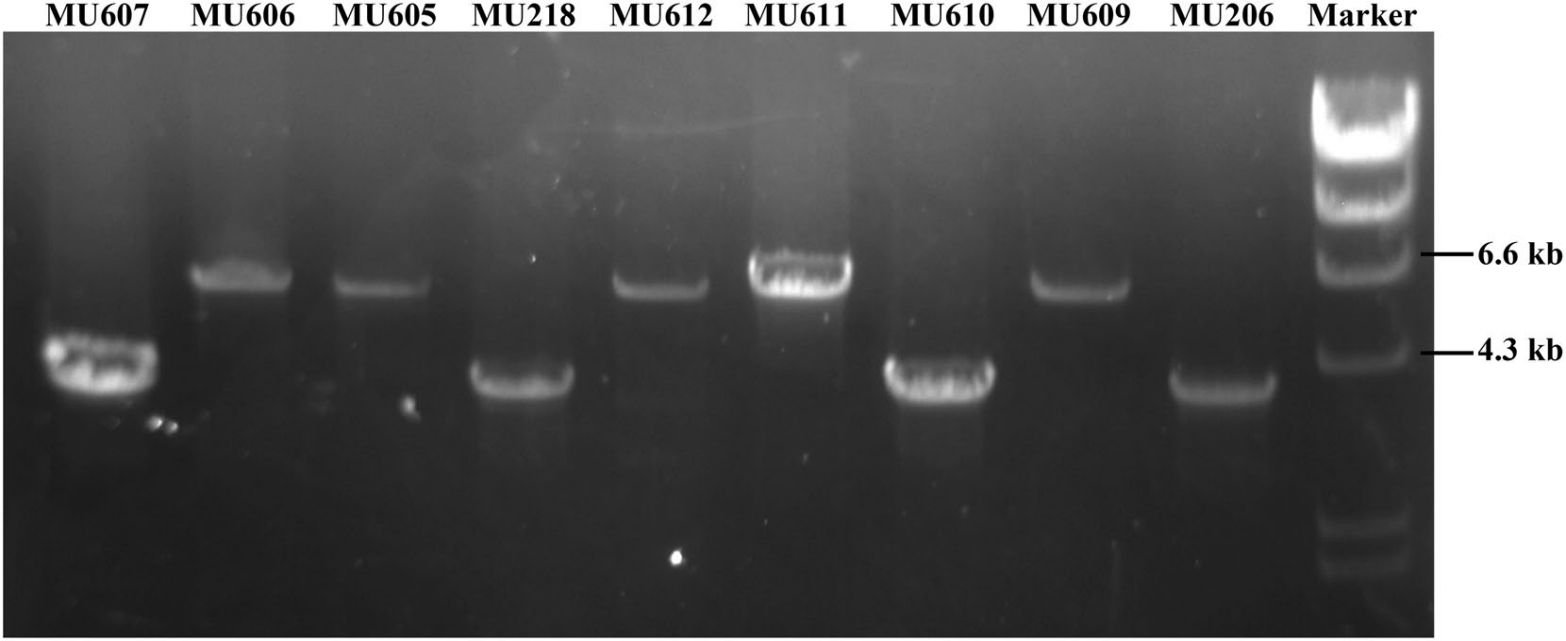
Analysis of the *crgA* locus in knockout mutants. Genomic DNA from the indicated strains was amplified by PCR using primers *cigC*-F1 and *crgA*-R1 that amplified wild-type (4.0 kb) and mutant *crgA* alleles (6.5 kb) (Fig. 1), and the PCR products were analysed by agarose gel electrophoresis. *MU605*, *MU606* and *MU607* were derived from *MU218*, whereas *MU609*, *MU610*, *MU611* and *MU612* were derived from *MU206*. *Marker*, λ DNA digested with *Hind*III. *Sizes* of the DNA marker relevant fragments are indicated on the *left side* of the figure

Transformation of MU206 and MU218 with the replacement fragment produced 27 and 31 transformants, respectively. The initial transformants were grown in a selective medium for several vegetative cycles to identify stable Leu^+^ transformants, which indicated that exogenous DNA integration had occurred. Finally, four and three homokaryotic transformants that showed a 100 % stable Leu^+^ phenotype were obtained from MU206 and MU218, respectively. Three transformants derived from MU206 (MU609, MU611 and MU612) presented a deeper yellow colour than the other (MU610). In a similar manner, two transformants derived from MU218 (MU605 and MU606) displayed a deeper yellow colour than the other (MU607). Colour differences could result from different integration events and, therefore, the genomic DNA of all seven homokaryotic transformants and the parental strains MU206 and MU218 were analysed by PCR using primers that amplify both the disrupted and the wild-type *crgA* locus-producing fragments of 4 and 6.5 kb, respectively. The transformants that showed the deepest yellow colour (MU605, MU606, MU609, MU611 and MU612) showed only the 6.5-kb PCR fragment (Fig. 2) that indicated their lack of a wild-type *crgA* allele, which had been replaced by the *leuA* gene (Fig. 1). In contrast, MU607 and MU610 showed the same 4-kb PCR fragment as the parental strains MU206 and MU218 (Fig. 2), which indicated that they contained a wild-type allele of *crgA* and therefore carried an ectopic integration of the replacement fragment.

### Analysis of carotenogenesis in *crgA*-null derivatives of MU206 and MU218

The effect of *crgA* deletion in mutants MU206 and MU218 was analysed by comparison of the β-carotene levels in the obtained transformants, the parental strains and a mutant with deletion of *crgA* in a wild-type background (MU221) [14]. The obtained transformants and MU221 were prototrophs, whereas the parental strains MU206 and MU218 were leucine auxotrophs. Due to the known effect of leucine on the carotenoid content [9], leucine auxotrophy of the parental strains was complemented by transformation with the autoreplicative plasmid pLEU4-producing strains MU608 and MU613, which were derived from MU218 and MU206, respectively (Table 4).

**Table 4:** β-carotene content in *crgA*-null derivatives of MU206 and MU218

Strain	Genetic background	*crgA* deletion	β-carotene(ug/g dry mass)	
			Dark	Light
MU241	Wild-type	−	25.9 ± 2.6^a^	667.3 ± 49.1^a^
MU221	Wild-type	+	1481.8 ± 131.0^c^	2003.1 ± 146.0^c^
MU605	MU218	+	2621.0 ± 204.0^f^	3559.0 ± 262.5^f^
MU606	MU218	+	3001.9 ± 232.1^g^	4009.2 ± 206.8^g^
MU607	MU218	−	93.0 ± 16.6^a^	1007.8 ± 40.0^ab^
MU608	MU218	−	97.2 ± 9.9^a^	1230.9 ± 54.1^a^
MU609	MU206	+	1817.6 ± 139.6^d^	2328.6 ± 152.9^cd^
MU610	MU206	−	441.1 ± 16.1^b^	1094.6 ± 47.4^ab^
MU611	MU206	+	2089.8 ± 117.6^e^	2879.0 ± 127.6^e^
MU612	MU206	+	1931.2 ± 97.9^de^	2591.6 ± 154.3^de^
MU613	MU206	−	661.3 ± 32.0^b^	1425.1 ± 51.4^b^

^a–g^Values are mean ± standard deviation of at least three independent experiments and values within a column with different superscript letters were significantly different (p < 0.05)

The β-carotene content both in the dark and in light in all *crgA* mutants derived from MU206 (MU609, MU611 and MU612) and from MU218 (MU605 and MU606) was higher than that seen in MU221 and the prototrophic MU206 strain (MU613). In contrast, the transformants that carried an ectopic integration of the replacement fragment (MU610 for MU206 and MU607 for MU218) showed β-carotene levels similar to those of the corresponding prototrophic strains (Table 4). Although the simultaneous presence of mutations in MU206 or MU218 and *crgA* deletion produced an increase in the β-carotene content compared to MU221, the effect was much greater with an MU218 background (Table 4).

**Fig. 3:**
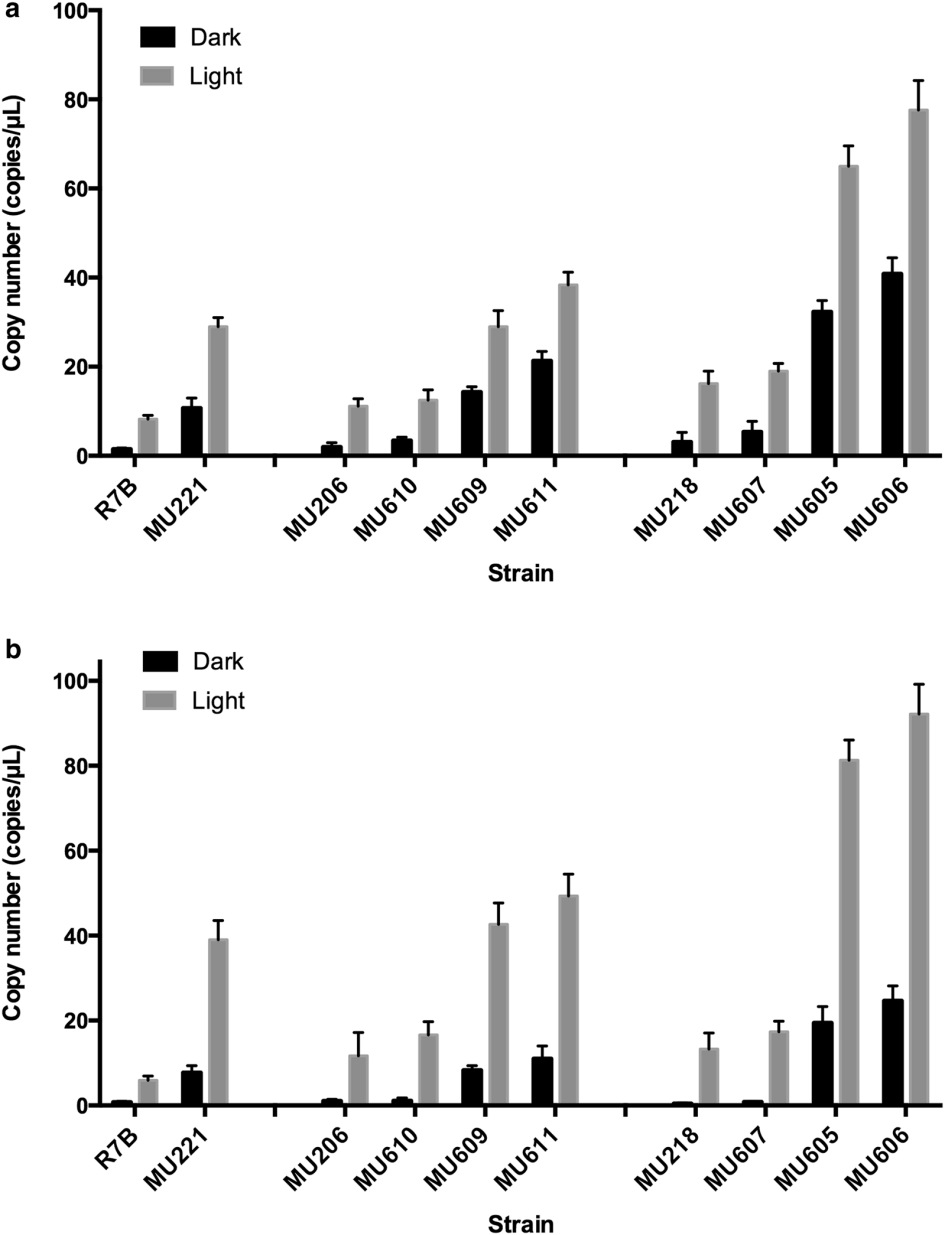
Expression of *carB* (**a**) and *carRP* (**b**) genes in *crgA*-null mutants derived from MU206 and MU218. Copy number of the *carB* (**a**) and *carRP* (**b**) mRNAs in total RNA sample from the indicated strains was determined by Droplet Digital PCR. The *carB* and *carRP* mRNA copy numbers were normalised to the actin reference gene. All RNA samples were diluted 10 times and detected in triplicate. *MU221*, prototrophic *crgA* mutant derived from *R7B*; *MU218* and *MU206*, derived from *R7B*, *leuA* ^−^; *MU605*, *MU606*: *crgA*-null mutants derived from *MU218*; *MU607*, prototrophic strain derived from *MU218*; *MU609* and *MU611*, *crgA*-null mutant derived from *MU206*; *MU610*, prototrophic strain derived from *MU206*

High levels of β-carotene can be produced by a high level of expression of structural carotenogenic genes [14]. To determine whether the high β-carotene levels in mutants derived from MU206 and MU218 were linked to a high level of expression of carotenogenic genes, the accumulation of *carB* and *carRP* mRNAs was analysed in mycelium grown both in the dark and in light by Droplet Digital PCR [29]. In agreement with the high carotenoid levels, mutants MU206 and MU218, and their corresponding strains with ectopic integrations, accumulated higher levels of *carB* and *carRP* mRNAs than the wild-type strain R7B after a light pulse, and also *carB* mRNA levels in the dark (Fig. 3). However, the *carB* and *carRP* mRNA levels in all *crgA*-knockout mutants were higher than those seen in any strain with a *crgA* wild-type allele. Thus, MU221 showed a considerable increase in the *carB* and *carRP* mRNA levels, in concordance with the high carotene levels accumulated by this strain. Also in agreement with the β-carotene content, the *carB* and *carRP* mRNA levels were increased in *crgA* mutants derived from strain MU206 (M609, and M611) and were increased even more in *crgA* mutants derived from MU218 (MU605 and MU606) (Fig. 3). Especially interesting was MU606, because it showed the highest *carB* and *carRP* mRNA levels, with 27-fold and sixfold increases in the *carB* mRNA levels and 25-fold and 15-fold increases in the *carRP* mRNA levels in the dark and light, respectively, with respect to the wild-type strain R7B. Moreover, it accumulated approximately 3 and 4 mg/g dry weight of β-carotene in the dark and in light, respectively. Compared to MU221, the β-carotene production in MU606 was doubled under both dark and light conditions. The disruption *crgA* gene in MU206 failed to improve the carotene production as significantly as in MU218. The β-carotene content of the transformants of MU206 is similar to that seen in MU221; even MU611, which possesses the highest amount of β-carotene amongst the M206 *crgA* mutants, rose slightly to 2.8 mg/g in light.

## Discussion

### New regulatory elements in the control of *M. circinelloides* carotenoid biosynthesis

Several partially characterised regulatory networks control carotenogenesis in fungi that belong to the mucoromycotina subphylum. In *P. blakesleeanus,* the biosynthesis of carotenoid biosynthesis is regulated by a complex regulatory network triggered with several environmental factors (blue light, sexual interaction, retinol and aromatic chemicals) that act in independent ways [30]. In *M. circinelloides,* two regulatory pathways have been identified that control different aspects of carotenoid biosynthesis [15]. Thus, the induction of carotenoid biosynthesis by light is mediated by the gene *mcwc*-*1c* [9], whereas the basal levels of carotenoids are under the control of a different regulatory pathway in which *crgA* and *mcwc*-*1b* are the main actors [14]. However, there should be an additional unknown regulatory mechanism, as suggested by the ability of light to stimulate carotene biosynthesis in *mcwc*-*1c*–null strains, although the effect is very weak [9].

The results of this work support the existence of additional regulatory mechanisms, because eight mutants were isolated that showed altered β-carotene levels even though they lacked mutations in the genes of the two previously known regulatory pathways. Five of the mutants (MU216, MU217, MU283, MU285 and MU286) showed reduced β-carotene levels only in light, which suggests that they are affected by a regulatory pathway involved in the induction by light of carotenoid biosynthesis. However, the absence of common mutations in the sequenced genomes of three of these mutants (MU216, MU217 and MU286) points to the existence of more than one regulatory pathway, although the notion that they carry mutations in different elements of the same pathway, even the *mcwc*-*1c* regulatory pathway [9], cannot be dismissed. The other three mutants (MU206, MU218 and MU264) showed alterations of β-carotene both in the dark and in light, although MU206 and MU218 retained their ability to respond to light, which indicates that they carry mutations in genes involved in mechanisms that control the basal carotenoid levels but not its induction by light. Interestingly, MU264 showed a complete blockage of carotenoid synthesis suggesting it defines a pivotal regulatory pathway that controls carotene biosynthesis. Moreover, the absence of shared mutations amongst these mutants suggests that they define either distinct regulatory pathways or different elements of the same pathway. In fact, the genes affected in these mutants, particularly mutants with high β-carotene levels (MU206 and MU218), could code for proteins that participate in the *crgA* regulatory pathway. The *crgA* gene represses carotenoid biosynthesis by proteolysis-independent mono-ubiquitilation and di-ubiquitilation of Mcwc-1b, which activates transcription of the structural carotenogenic genes *carB* and *carRP* when it is non-ubiquitilated [15]. As a result, deletion of *crgA* led to significant improvement in carotene accumulation even though carotenogenesis was still induced by light [14]. The β-carotene content in the mutants MU206 and MU218 somehow resembled the phenotype of the *crgA*-null mutant MU221, which suggests that they could be affected in genes that participate in the same regulatory pathway as the *crgA* gene. In fact, MU206 and MU218 also showed higher mRNA levels of the structural carotenogenic genes than the wild-type strain (Fig. 3), which suggests that the mutations in these mutants increase the transcription of structural carotenogenic genes in a fashion similar to that of loss-of-function mutations of *crgA* [14].

To gain insight into the underlying regulatory mechanism altered in mutants MU206 and MU218 and its relationship with the *crgA* gene, this gene was disrupted in both MU206 and MU218. Disruption of the *crgA* gene in those mutants resulted in enhancement of both the mRNA levels of carotenogenic genes (*carB* and *carRP*) and the β-carotene content in comparison to the recipient strains. Moreover, both MU206-derived and MU218-derived mutants were able to increase both mRNA levels of carotenogenic genes and β-carotene content in response to light, which indicates that the regulatory mechanism that controls induction by light was operating in both types of mutants. However, the expression enhancement of carotenogenic genes in the MU218-derived mutants was much greater than that in the MU206-derived mutants both in the dark and in light, and it was linked to significantly greater accumulation of β-carotene. In fact, the mRNA levels of carotenogenic genes in the *crgA*-null mutants derived from MU218 greatly exceeded those shown by MU221 both in the dark and in light (Table 4). Even more interesting was that they doubled the amount of β-carotene present in MU221 both in the dark and in light. These results suggest that some of the mutations in MU218 affect one or some of the genes that control the expression of carotenoid genes independently of *crgA*, thus revealing the existence of an additional mechanism involved in the regulation of carotenoid biosynthesis in *M. circinelloides*. This mechanism should repress the expression of carotenoid structural genes to a lesser extent than *crgA* because mRNA levels of carotenogenic genes and carotenoid content are much lower in MU218 than in MU221, particularly in the dark, where there is no interference by light stimulation (Table 4). Therefore, these two regulatory mechanisms may work in parallel, and the mutants for both mechanisms showed consequently higher expression of carotenogenic structural genes and β-carotene content than mutants for just one mechanism. Unfortunately, the presence in MU218 of mutations in several genes renders analysis of the identified regulatory mechanism impossible at this time. Further work is necessary to identify the gene responsible for the phenotype of MU218 and to understand the manner in which it regulates carotenoid biosynthesis.

In contrast, although *crgA* mutants derived from MU206 also showed higher levels of both mRNAs of carotenogenics genes and β-carotene than MU221 both in the dark and in light, the increases were much more discrete than those seen in the *crgA* mutants derived from MU218 (Fig. 3; Table 4). Although the existence of a fourth regulatory pathway in the control of carotenogenesis, which is affected in MU206, cannot be discarded, it is more probable that the gene responsible for the MU206 phenotype participates in the *crgA* regulatory pathway. This idea is supported not only by the discrete increase in expression of carotenogenic genes and carotene content observed in the *crgA* mutants derived from MU206 over MU221, but also by the similarity in the patterns of β-carotene accumulation shown by MU206 and MU221 (Table 4). Therefore, it is tempting to speculate that MU206 carries a mutation in a gene that acts upstream of *crgA* or participates with *crgA* in the repression of carotenogenic structural genes. Further experiments are required to identify the gene responsible for the phenotype of MU206 and understand the relationship with *crgA* in the regulation of carotenogenesis.

### A putative candidate for industrial production of β-carotene

As one of the essential pigments, β-carotene is the precursor of vitamin A, retinal and retinoic acid, which are of critical importance in vision, nutrition and cellular growth [31–33]. β-carotene biosynthesis has been described extensively in the zygomycetes *B. trispora*, *P. blakesleeanus* and *M. circinelloides*, which are promising organisms for industrial production. Compared to the other two fungi, *M. circinelloides* could be an interesting alternative as a biotechnological β-carotene source because of the efficient tools available for genetic manipulation. This work represents a proof of concept of this idea because the disruption of the *crgA* gene in mutants (MU206 and MU218) that are affected in the regulation of carotene biosynthesis generated several β-carotene–overaccumulating strains. Amongst these strains, the largest producer of β-carotene, MU606, accumulated 3001.9 ± 103.7 and 4009.23 ± 180.4 μg/g β-carotene of dry mass in the dark and in light, respectively. Therefore, it yielded approximately five and two times more β-carotene than the wild-type strain and MU221. Despite these increases in β-carotene levels, the amount of β-carotene in this strain is far from the main current industrial source of β-carotene [34], *B. trispora,* which is capable of producing 30 mg/g [35] by mixing two strains of opposite mating types in an optimised enrichment medium. Industrial β-carotene production using *M. circinelloides* could be simpler than the complicated fermentation process used with *B. trispora*. In addition, the yeast-like morphology of this dimorphic fungus facilitates biotechnological production [36]. Therefore, the β-carotene–overaccumulating strain obtained in this study is a strong putative candidate for industrial production of β-carotene because additional genetic manipulation or optimisation of growth conditions could generate improved strains comparable to industrial strains of *B. trispora*.

## Conclusions

In conclusion, we adopted a classical forward strategy combined with modern reverse genetic techniques to deepen the regulation of carotenoid biosynthesis and the generation of strong candidate strains of industrial interest for β-carotene production. The results suggest the existence of a new regulatory mechanism that represses carotenoid biosynthesis independently and in parallel to the previously known repressor *crgA*. Like crgA, it acts at the level of mRNA accumulation keeping the transcript levels of carotenogenic genes low. Although the key gene involved in this new regulatory mechanism is pending on determination, a set of candidates has been identified by whole-genome sequencing. In addition, a double mutant for *crgA* and the new identified regulatory element was capable of accumulating up to 4 mg/g of β-carotene, being a candidate to be target of further improvements for biotechnological β-carotene production.

## Methods

### Strains, plasmids and transformation conditions

The strains generated in this work were derived from R7B, which is a leucine auxotroph of *M. circinelloides* f. *lusitanicus* CBS277.49 [37] that keeps a wild-type phenotype for carotenoid biosynthesis [25]. Spores of this strain were mutagenized with UV light (50 mJ/cm^2^) or NTG in conditions that produced a 1–5 % survival rate as previously described [25]. Due to the multinucleate state of R7B spores, spores were allowed to complete a vegetative cycle in complete medium to unveil the expression of recessive mutations. To reduce the probability to isolate the same mutant several times, spores to be mutagenized were split in different tubes and kept separately after recycling.

The strains MU241 and MU221 were also derived from R7B; MU221 is a *crgA*-null mutant, and MU241 is a prototrophic wild-type strain after replacement of the previous *leuA* mutant allele with the wild-type allele [14]. Plasmid pLEU4 [37], which is an auto-replicative plasmid that harbours a wild-type *leuA* allele, was used to complement the *leuA* auxotrophy of the recipient strains. Plasmid pVEN172 [14] that contains a 6.5-kb *Pst*I fragment of the wild-type allele of *leuA* gene flanked by DNA regions adjacent to the *crgA* gene was used to disrupt the *crgA* gene of MU206 and MU218. Cultures were grown in minimal medium (YNB) [38] or complete medium (YPG) [39]. l-leucine (20 μg/ml) was supplied when required. The transformation procedure has been described previously [40].

### Nucleic acid manipulation and analysis

Genomic DNA of *M. circinelloides* was prepared as described previously [41]. Plasmid was isolated by the rapid boiling method [42] and purified following the manufacturer’s instructions (Thermo Scientific, USA). The presence of mutations in carotenogenic genes in the mutants was analysed by PCR amplification of *carB*, *carRP*, *mcwc*-*1c* and *mcwc*-*2* genes (ID78608, ID85699, ID153812 and ID157130) using the primers listed in Additional file 7: Table S5 and PfuUltra II Fusion HS DNA Polymerase (Agilent). The PCR fragments were directly sequenced without cloning to avoid interference with mutations produced during PCR, and the obtained sequence was compared to the reference genome of *M. circinelloides* CBS277.49 (http://www.genome.jgi.doe.gov/Mucci2/Mucci2.info.html).

Linear DNA fragments for transformation were released by treating the plasmid pVEN172 with *Pst*I, and then purified from agarose gel with GeneJET Gel Extraction Kit (Thermo Scientific, USA). To determine whether the fragment was integrated into the genome correctly, PCR amplification in an “Eppendorf Mastercycler personal” was performed with genomic DNA as a template and Herculase II fusion DNA polymerase (Agilent Technologies, USA) following the manufacturer’s instructions. Primers *cigC*-F1 and *crgA*-R1 were designed for amplification of the recombinant fragment (Additional file 7: Table S5). The samples were incubated at 95 °C for 3 min for pre-denaturation and subjected to 30 cycles of denaturation (95 °C for 30 s), annealing (55 °C for 30 s) and extension (72 °C for 3 min 30 s) with an additional 5-min extension step.

The transcription level of the structural genes *carB* and *carRP* was analysed with the Droplet Digital PCR technique (ddPCR). Total RNA was isolated using Trizol reagent following the instructions of the manufacturer (Invitrogen). Reverse transcription was performed with PrimeScript RT Reagent Kit with gDNA Eraser (Takara), and the ddPCR experiments were carried out with QX200™ ddPCR™ EvaGreen Supermix (BioRad). The PCR reaction mix was partitioned into water-in-oil emulsion droplets with a QX100 Droplet Generator. The droplets were then transferred to a 96-well PCR plate and put into a thermal cycler (Bio Rad C1000). Thermal cycling conditions were [95 °C × 5 min; 40 cycles of (95 °C × 0.5 min, 60 °C × 1 min); 4 °C × 5 min; 98 °C × 5 min (ramp rate set to 2 °C/s)]. The PCR plate was then moved to the QX100 Droplet reader for automatic reading of samples. The copy numbers of *carB* and *carRP* genes from various samples were normalised to the actin reference gene (ID157034; scaffold_07: 2052804–2054242).

For whole-genome sequencing of the DNA of carotenogenic mutants, genomic DNA was isolated by cesium chloride gradient [43], sequenced using SOLiD technology and analysed at the Centre for Genomic Research in the University of Liverpool. A sample of mutations was validated by direct sequencing of PCR products from mutated genes in strains MU206, MU264 and MU286 using specific primers (Additional file 7: Table S5). The sequence reads have been deposited in the European Nucleotide Archive and the accession number is PRJEB13169.

### Analysis of carotenes

Carotenes were extracted from mycelia grown on YNB solid medium or YNB supplemented with leucine for leucine auxotrophic strains, for 84 h in the dark and 60 h in the dark followed by 24 h in light at 26 °C. The mycelia were washed and dried between paper towels and triturated with a pestle in the presence of liquid nitrogen. After lyophilisation, 2 mg of the freeze-dried mycelium powder was weighed, suspended in 2 ml of methanol and extracted several times with 2 ml of petroleum until the mycelium was colourless [44]. For spectrophotometric quantification of β-carotene, the absorption coefficients given by Davies [45] were applied.

To HPLC analysis of the carotenoids, the organic phase extracted as described above were mixed and evaporated at 30 °C in vacuum. All samples were suspended in 500 µL ethyl acetate and filtered (0.22 μm) prior to HPLC with diode-array detection HPLC–DAD analysis. Carotenoids were identified and quantified using a column ZORBAX Eclipse XDB-C18 end capped 5 μm, 4.6 × 150 mm reverse phase column (Agilent Technologies, USA). The eluents used were (A) acetonitrile: water (9:1, v/v) and (B) ethyl acetate. The column separation was allowed via a series of gradient such as follows: 0–40 % solvent B (0–20 min), 40–60 % solvent B (20–25 min), 60–100 % solvent B (25–25, 1 min), 100 % solvent B (25, 1–35 min) and 100–0 % solvent B (35–50 min) at a flow rate of 1 ml/min. The injection volume was 15 μl each one. Detection of individual carotenoids was made at 284 and 453 nm, which are the wavelength of maximum absorption of phytoene and β-carotene. These compounds were identified by co-chromatography with standards and by elucidation of their spectral characteristics using a photo-diode array detector. The concentrations of carotenoids were estimated on basis of an adequated standard curve using their respective standard compounds [46].

### Statistical analysis

All experiments were carried out independently at least three times, and the mean values ± SD were presented. Statistical analysis was performed with one-way analysis of variance with SPSS 20.

## Additional files

10.1186/s12934-016-0493-8 Spectrophotometric scans from 350 nm to 550 nm of total carotenoid extracts from the indicated strains. Absorbance scales are different in each sample.
10.1186/s12934-016-0493-8 HPLC elution profile of carotenoids accumulated by the indicated strains in light. Carotenoids were detected at 453 nm.
10.1186/s12934-016-0493-8 Coverage statistic of whole-genome sequencing.
10.1186/s12934-016-0493-8 Summary of SNPs identified in the genomes of carotenogenic mutants.
10.1186/s12934-016-0493-8 SNPs identified in the genomes of carotenogenic mutants.
10.1186/s12934-016-0493-8 Indels identified in the genomes of carotenogenic mutants.
10.1186/s12934-016-0493-8 Primers used in this study.
