# Supplementary material for: A new regulatory mechanism controlling carotenogenesis in the fungus Mucor circinelloides as a target to generate β-carotene over-producing strains by genetic engineering
Source: Microb Cell Fact. 2016 Jun 7;15:99. doi: 10.1186/s12934-016-0493-8 (PMC4897934; doi:10.1186/s12934-016-0493-8)
Supplement: Supplementary file 1 — 10.1186/s12934-016-0493-8 Spectrophotometric scans from 350 nm to 550 nm of total carotenoid extracts from the indicated strains. Absorbance scales are different in each sample. [file 12934_2016_493_MOESM1_ESM.pptx]

## Slide 1
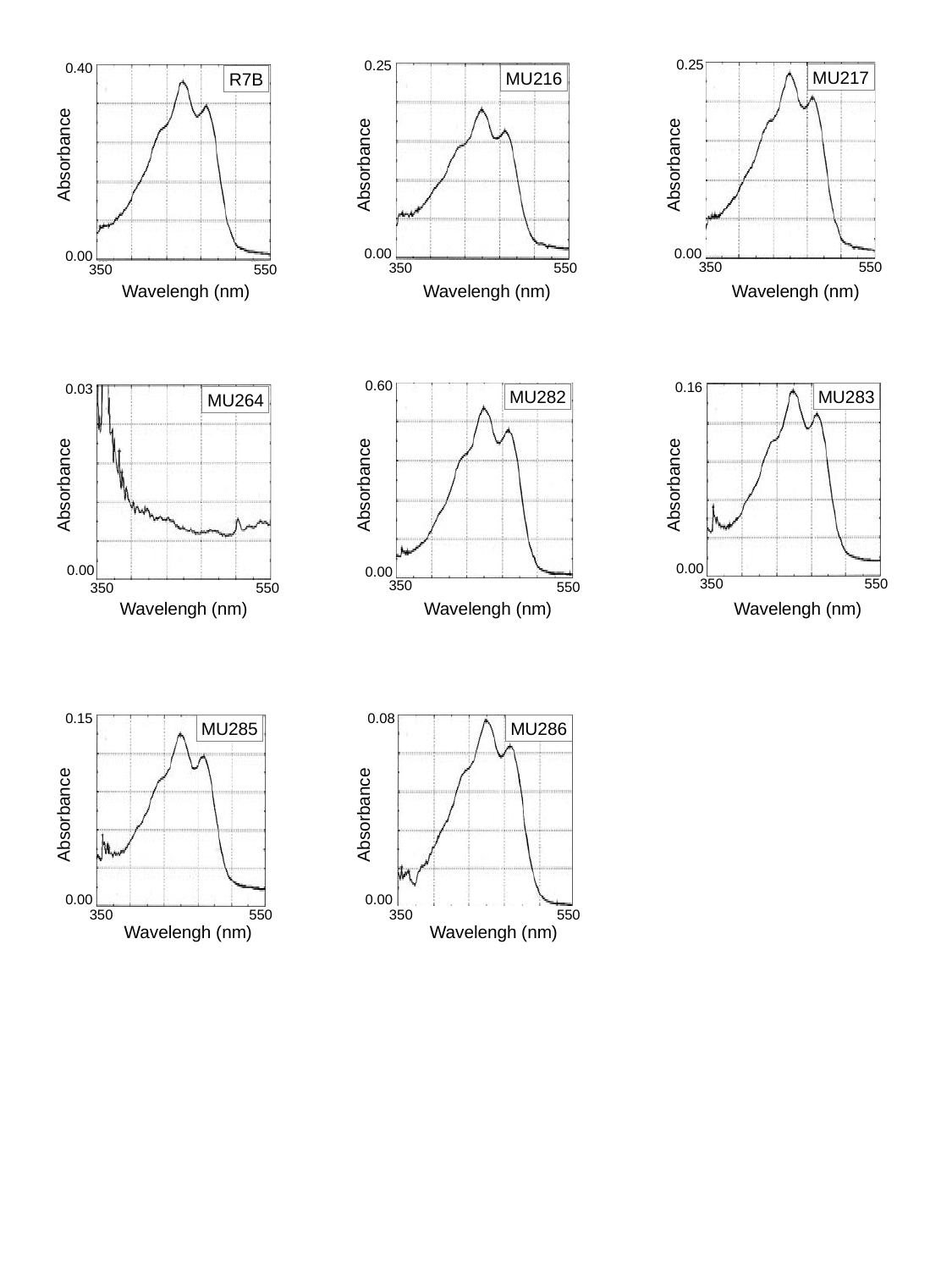

0.25
0.25
0.40
MU217
MU216
R7B
Absorbance
Absorbance
Absorbance
0.00
0.00
0.00
350
550
550
350
350
550
Wavelengh (nm)
Wavelengh (nm)
Wavelengh (nm)
0.60
0.16
0.03
MU282
MU283
MU264
Absorbance
Absorbance
Absorbance
350
550
350
550
350
550
Wavelengh (nm)
Wavelengh (nm)
Wavelengh (nm)
0.00
0.00
0.00
0.15
0.08
MU285
MU286
Absorbance
Absorbance
350
550
350
550
Wavelengh (nm)
Wavelengh (nm)
0.00
0.00
