# Supplementary figures and images for: A new regulatory mechanism controlling carotenogenesis in the fungus Mucor circinelloides as a target to generate β-carotene over-producing strains by genetic engineering
Source: Microb Cell Fact. 2016 Jun 7;15:99. doi: 10.1186/s12934-016-0493-8 (PMC4897934; doi:10.1186/s12934-016-0493-8)

## Slide 1
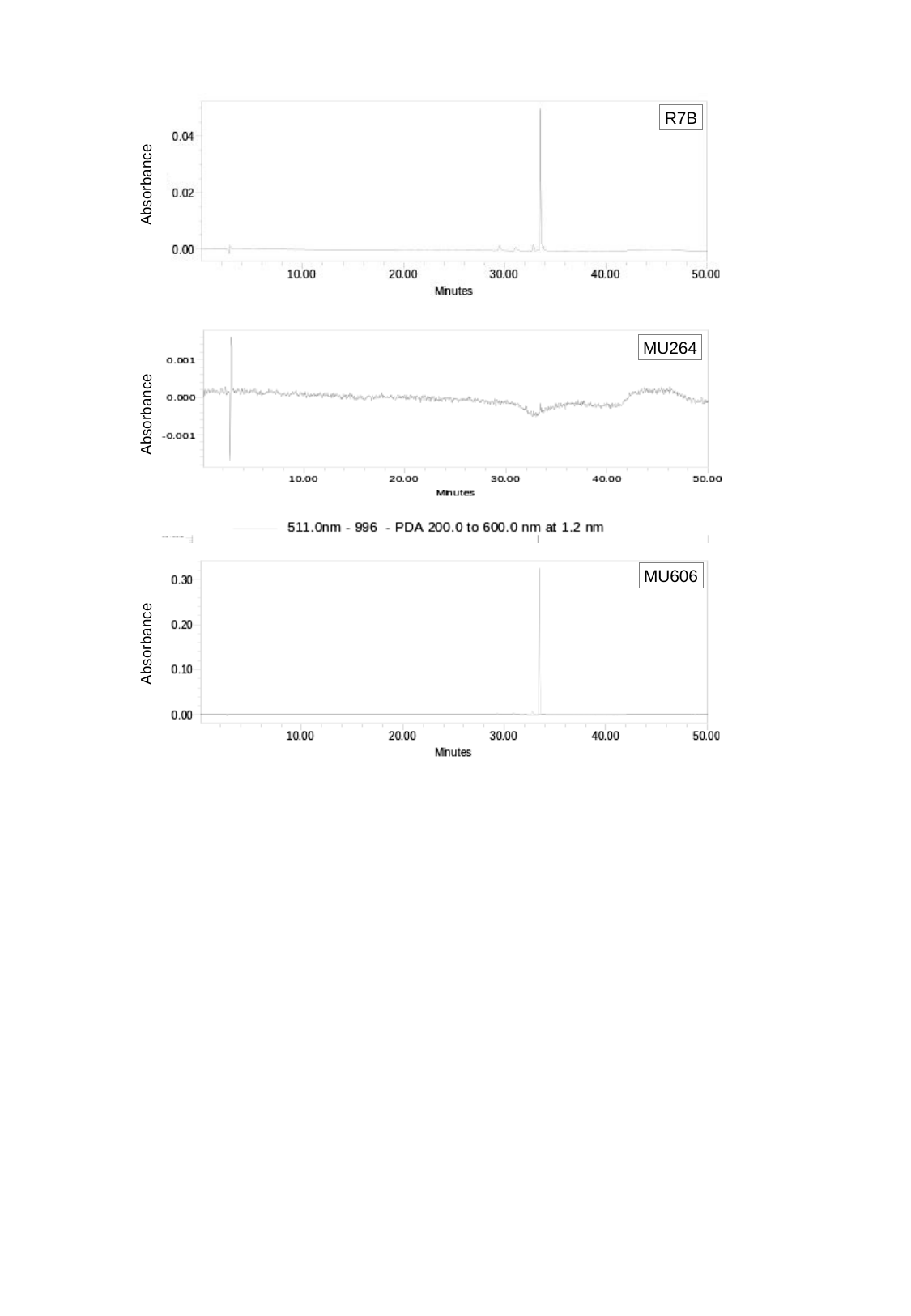

R7B
Absorbance
MU264
Absorbance
MU606
Absorbance

Supplement: Supplementary file 2 — 10.1186/s12934-016-0493-8 HPLC elution profile of carotenoids accumulated by the indicated strains in light. Carotenoids were detected at 453 nm. [file 12934_2016_493_MOESM2_ESM.pptx]
