# Supplementary material for: A new regulatory mechanism controlling carotenogenesis in the fungus Mucor circinelloides as a target to generate β-carotene over-producing strains by genetic engineering
Source: Microb Cell Fact. 2016 Jun 7;15:99. doi: 10.1186/s12934-016-0493-8 (PMC4897934; doi:10.1186/s12934-016-0493-8)
Supplement: Supplementary file 3 — 10.1186/s12934-016-0493-8 Coverage statistic of whole-genome sequencing. [file 12934_2016_493_MOESM3_ESM.doc]

Table S1 Primers used in this study

| Primers | Sequence (5´→3´) | Gene |
| --- | --- | --- |
| *cigC*-F1 | TATCCTCGCTGGACAACCGTGC | *crgA* |
| *crgA*-R1 | ACAACCCCTTTGCCATTCATCC |
| dd*actin*-F | TTCCTATGTTGGTGATGA | *actin* |
| dd*actin*-R | GTTGTAGAAGGTGTGATG |
| dd*carB*-F | AAGATTAACACCGATGATG | *carB* |
| dd*carB*-R | AGATTGTGATAAGCATAGAC |
| Pp1-for2 | AACTGCGGGCCCGTCGACCCTGGTTGATGTTCTTATC | ID107362 |
| Pp1-rev1 | CCAAGAGCACCGCCAACG |
| Fbox-for1 | CTCGCAGGGCCCCGAGCGTACAAAAAAGTCATC | ID166227 |
| Fbox-rev2 | TGTGTTGCTGGGCCCGTTGTTTGGCTGAGTGCTGC |
| Agc1-for2 | GCAGAGGAGTATTCAACAGC | ID81312 |
| Agc1-rev4 | AGCACCGATGATGTGGATGA |
| Agp9-for2 | CTGTCCTTCTGCTTGCTGG | ID116964 |
| Agp9-rev4 | GAAAAGGGCCCTTTCGGGAGTTACCTCCATG |
| vpsA-for1 | GCAGCGGGCCCCTCCTCAACAGCAACAGGCAC | ID157796 |
| vpsA-rev1 | CTCACCATGTTCAACTTGCG |
| lp3-for2 | CTGCCTCTTGGCGATTGAATC | ID111734 |
| lp3-rev2 | TGCGCGGGCCCAAAGAGACATCCACCTCAGC |
| sep1-for1 | GTCTTGGGTACCCAAATGAATATATAAAGGGTC | ID104636 |
| sep1-rev1 | GCGTTTCTCGAGGTCAATCATGGAAAGAAGG |
| *carRP*4 | AAGAGCTTGTCGACTATTTAGATTTCTCATTTTTCC | *carRP* and *carB* |
| *carB*3 | AGTTAAGGGAGTTAGTGCTAG |
| *mcwc-1c*-p27 | GACCGTCGAGAATGTTGCAC | *mcwc-1c* |
| *mcwc-1c*-p8 | TACATTAATGGTAGGACT |
| *mcwc-2a*-p1 | CTTGGGAAAGGAGAGAGCATAC | *mcwc-2a* |
| *mcwc-2a*-p2 | TGTTGATGACGAAATGTTAATTAG |
| *mcwc-2b*-p1 | GCGCTATGTTTATATGCTTGATTC | *mcwc-2b* |
| *mcwc-2b*-p2 | AGAATACCAAGATAATGTAATTG |
| *mcwc-2c*-p1 | TGATTACCATAGCAACAAATC | *mcwc-2c* |
| *mcwc-2c*-p2 | GTATAATATGATGAGAGAATCTTGGT |
| *mcwc-2d*-p1 | ACACCAGCGAGCAGGACGACG | *mcwc-2d* |
| *mcwc-2d*-p2 | GTGATCATTTCAATAAAACGC |
